# Changes in the selected reproductive health indicators among married women of reproductive age in low performing areas of Bangladesh: findings from an evaluation study

**DOI:** 10.1186/1471-2458-14-478

**Published:** 2014-05-21

**Authors:** Rukhsana Gazi, Humayun Kabir, Nirod Chandra Saha

**Affiliations:** 1 Centre for Equity and Health Systems, icddr,b, Dhaka, Bangladesh

**Keywords:** Reproductive health, Married women, ANC, CPR, Family planning, Awareness, RTI/STI, Pregnancy complications, Bangladesh

## Abstract

**Background:**

Three-year duration Demand-Based Reproductive Commodity Project (DBRHCP) was launched in three low performing areas: rural Nabiganj (population 323,357), Raipur (population 260,983) and urban slum in Dhaka (population 141,912). Objectives: Assessing changes in knowledge among married women of reproductive age on selected reproductive health issues and to explore their service utilization patterns over the project period in selected low performing areas of Bangladesh.

**Methods:**

The study adopted a pre- posts design. In the project areas, the entire chain of service provision were modified through the interventions under the DBRHCP, including training of the providers, enhanced behavioral change communication activities, follow-up and counseling, record keeping, reporting and monitoring, as well as improvement in logistics and supplies. Peer promoters were established as linkages between clients and service providers. All households were enlisted. Baseline and end line surveys were done using representative simple random sampling method, capturing changes over one year intervention period. Descriptive analysis was done using SPSS package, version 10. Proportional tests using Stata, version 8 were done to assess changes from baseline to end line.

**Results:**

The overall contraceptive prevalence was markedly different in the three study areas but significantly increased in both Dhaka urban slums and Nabiganj. In the rural areas, a higher proportion of the women in endline compared to baseline obtained contraceptive methods from the public sectors. Irrespective of study sites, significantly higher proportion of women received ANC (Antenatal Care) and PNC (Post natal care) in endline compared to baseline. In all study sites higher proportions of women were aware of maternal complications at endline. Services were obtained from qualified persons for reported symptoms of sexually transmitted infections by a higher proportion of women at endline compared to baseline. There were improvements in other RH indicators, such as use of skilled birth attendants and overall utilization of health care facilities by women.

**Conclusions:**

The improvements in several important RH indicators in the intervention areas suggest that the interventions affected selected outcomes reported in the study. The study findings also suggest that investment in the reproductive health sector, particularly in existing government programs, improves RH outcomes.

## Background

Bangladesh had a population density of 1142.29 in 2010, according to a World Bank report published in 2012 [1]. One of the challenges in Bangladesh is to achieve replacement level fertility. To achieve a replace level fertility (on average a woman should have 2.1 children to replace herself and her mate), there must be an increase in users of permanent and semi-permanent contraceptive methods. Previous family planning and reproductive health programs in Bangladesh have traditionally been supply-oriented; they aimed to provide the means of effective contraception and family planning. This strategy was remarkably successful in the 1980s and early 1990s when the total fertility rate declined rapidly [2]. However, since 1998 there has been little decline in fertility [2]. This slow decline in the total fertility rate exposed the limitations of supply-oriented system. In addition to the problem of stagnant or slow fertility decline, reproductive health in Bangladesh faced other challenges, one of the most important being the lack of acceptance of different types of available methods leading to discontinuation of method use. A study conducted in rural Bangladesh reported that discontinuation of oral pill user was 43% and the commonest reason was perceived side effects [3].

Literature review shows that utilization of ante natal care (ANC) services by women in Bangladesh is increasing but still it is low [4,5]. Many women in Bangladesh consider pregnancy as normal event unless complications arise and thus they refrain from obtaining routine care [6]. Secondary analysis of BDHS data identified strong urban–rural differentials in receiving ANC and PNC (postnatal care) from medically trained providers [4,7]. For instance, Rahman [7] reported that urban mothers receive more PNC (77%) from medically trained providers compared to their rural counterparts (58.6%). The study shows that women who received PNC had a history of pregnancy complications and had received ANC from qualified providers [7]. In Bangladesh, the majority of births are still taking place with unskilled attendants in household settings and this is particularly true of women from lower socioeconomic status [8]. A study among ultra poor households in Bangladesh has reported that low parity, residence in the urban areas, higher educational attainments, and higher economic status were associated with utilization of trained personnel at the time of delivery [9]. A national level survey confirmed that many women in Bangladesh have low level of knowledge about maternal complications [2]. In a previous study done in Bangladesh emphasized that financial constraints, coupled with traditional beliefs and rituals, delayed care seeking considerably in cases of obstetric complications [9].

Sexually transmitted infections (STI) has adverse consequences on maternal and neonatal health including infertility, pelvic inflammatory diseases, ectopic pregnancy, cervical cancer, fetal wastage, low birth weight, infant blindness, and pre term births [10]. Married women in Bangladesh are at risk of acquiring STIs, for instance a study done among Bangladeshi women identified unexpected high prevalence of herpes simplex type 2 infection [11]. Socially, a lack of awareness and cultural taboos can increase woman’s risk of contracting RTI/STI due to unsafe behavior and then inhibit them from discussing their problems and seeking appropriate treatment. For example, a review found that often women do not view RTI/STIs as purely a biomedical problem, but blamed it on the larger stresses in their lives, social and economic [9]. The review also found that treatment for STI problems was sought mostly from female relatives and friends, healers, homeopaths, pharmacists and the least from allopathic doctors as it is culturally undesirable for women to be seen and be physically examined by male providers [9].

The National Institute of Population Research and Training (NIPORT) under the Ministry of Health and Family Welfare (MoHFW) of the Government of Bangladesh launched the three-year Demand-based Reproductive Commodity Project (DBRHCP) in July 2005 in two rural sub-districts and one urban slum areas [12]. This article is presenting part of the findings from evaluation of this large project. The specific objective of the present study was to assess changes in knowledge among married women of reproductive age on selected reproductive health issues and to explore their service utilization patterns over the project period in selected low performing areas of Bangladesh.

## Methods

The study was implemented in three low performing areas [13] of Bangladesh which included four wards of Dhaka City Corporation and two rural upazilas (sub-districts) namely Nabiganj (Northern, 192 km from capital Dhaka) and Raipur (Southern, 135 km from Dhaka). The Population Council, Research Training and Management International, John Snow International/Deliver Bangladesh and icddr,b (International Centre for Diarrhoeal Diseases Research, Bangladesh) were involved in implementation of this project. At the beginning of the project needs assessments were done, both at facility and community level, to design the intervention strategies.

Under the DBRHCP, the existing government service providers were trained (37 in Nabiganj and 36 in Raipur) on quality service provision and identification of unexpressed needs. Service providers received four-day training on contraceptive methods and another five days training on syndromic management of STIs. Field workers were given three-day training (25 in Nabiganj and 21in Raipur area) on FP counseling. Behavior change communication materials were developed for use during service provision. Equipment for developing quality FP and RH services and regular supplies of contraceptives had been ensured. Peer promoters were introduced at the community level to act as health promoters and also built referral linkages between community and the providers. Issue based street drama were conducted at market places to increase awareness in the community, particularly on family planning, maternal health, pregnancy complications and utilization of health care. A total of 10 dramas were staged where approximately 300 community members enjoyed the drama each time. The health systems were made more accommodative to females and males; couple counseling was promoted. Couples were rewarded for limiting their families and utilizing family planning services. Community support groups (CSG) were established in each union in order to ensure community involvement in the intervention activities, comprised of both male and female key person from the locality who attended monthly meetings. The members of CSG were oriented to build awareness in the community and collaborate with intervention activities. They monitored the progress of the interventions. Nine CSG meetings (one for each CSG) were held each month in each union. It was anticipated that the entire chain of service provision would be improved over the project period, including service delivery, follow-up and counseling, record keeping, reporting and monitoring, as well as logistics and supplies.

At the beginning of the project icddr,b conducted an enumeration of all households in the three project areas using locally recruited enumerators. Enumeration was done in three areas: slums of Dhaka city with a population of 141,912; one rural sub-district in Sylhet Division in the north of the country with a population of 323,357; and another rural sub-district in Chittagong Division in the south of the country with a population of 260,983. This provided socio-economic and demographic information as a basis for targeted interventions, and a sampling frame for the baseline survey. After household, enumeration a baseline was done during November 2006 to March 2007. Endline was conducted during November 2008 to March 2009. There was a 18 months intervention period between baseline and end line surveys.

The project was targeted to currently married women of reproductive age (MWRA), their husbands, and their adolescent daughters. This article only highlights the changes among married women on selected reproductive health indicators over the project period as a part of evaluation of the large project. The study population included currently married women (13–49 years) living in the three project sites. According to the enumeration data, 54,116 married women were living in Nabiganj Upazila, 49,585 in Raipur Upazila and 29,904 in urban slum areas of Dhaka city.

To estimate the required sample size we have considered current rates of selected method specific CPR (contraceptive prevalence rate), ANC, PNC, and delivery by trained birth attendants (BDHS, 2004) and we anticipated 5 to 7% changes from baseline to end line. The samples have been calculated at 95% confidence intervals with 90% power. The required sample size for selected indicators we proposed to have 7000 sample households in each of rural area and 6000 in urban area. We selected MWRAs from the household enumeration list by simple random selection procedure. The total sample would allow 5% non-response in rural areas and 10% non-response in urban area. Three subsequent attempts were made to cover absentees.

Women were interviewed using a structured questionnaire applied by 54 trained female interviewers both at baseline and end line. The study population was same at baseline and end line, but the sample was drawn separately at two different points. Nonresident women who visited from other areas during data collection were excluded. The interviewers were familiar with different aspects of the project objectives, interventions, and outcomes including issues on sexuality, family planning, STIs and reproductive health needs of male and female. The issues covered in the training were contraceptive methods, pregnancy complications, danger signs, service seeking behaviour and access to service centers, and mode of transmission of STIs. Each day after returning from the field, the interviewers crosschecked the completed questionnaires. The field supervisors reviewed each of the questionnaires and conducted regular spot-checking to maintain data quality. An experienced field research manager coordinated the overall field activities. Non response rate was 3% in urban and 2.7% in rural areas.

The study was approved by the Research Review Committee and Ethical Review Committee of icddr,b. Written informed consents for participation in the study was obtained from all respondents (adult females) of the study. Data was entered in Visual Foxpro, version 6 and descriptive analysis was done using SPSS computer packages, version 10. Data consistency was checked and data coding was done. Descriptive analysis was done to see status of selected indicators among MWRA both at baseline and end line. To assess statistical significant difference between baseline and endline in selected indicators in each area, proportional test was applied and p values (p < .001 at 95% level) were obtained using Stata version 8.

## Results

### Socio-demographic characteristics of the respondents

Table 1 shows that age distribution of MWRAs were similar in baseline and end line surveys. In general, more than 50% of respondents were aged 20 to 34 both in baseline and end line. The proportion aged 15–19 was highest in Dhaka and lowest in Nabiganj. Overall educational attainment of the respondents was low in all areas irrespective of two surveys. Above 48 (base line) to 47% (end line) respondents in Nabiganj and 48 to 45% in Dhaka had no formal education compared to 30 to 33% in Raipur. The proportion of women having completed secondary education or higher were lowest in Nabiganj (2.8 to 27%) and highest in Raipur (5.6 to 65%). In both rural and urban areas, the majority of women were housewives both in baseline and end line surveys. However while 94 to 95% of women were housewives in rural areas, only 72% were in Dhaka. Among the women in urban slums, 10 to 11% worked as housemaids and another 5 to 6% were involved in the garment industry.

**Table 1:** Distribution of Socio-demographic characteristics of surveyed women by study areas

**Age in years**	**Dhaka %**		**Raipur %**		**Nabiganj %**	
	**Base n = 5893**	**End n = 6026**	**Base n = 6795**	**End n = 6602**	**Base n = 6983**	**End n = 7009**
≥14	0.2	0.2	0.1	0.0	0.0	0.0
15-19	10.1	8.7	8.8	6.9	6.6	3.7
20-24	23.1	23.3	18.9	17.7	17.2	15.0
25-29	22.4	23.2	19.6	20.3	19.7	20.9
30-34	14.2	16.3	16.0	15.5	16.8	18.0
35-39	13.3	13.8	13.8	14.0	14.7	17.5
40-44	8.9	8.4	12.6	11.4	12.9	12.6
45-49	7.9	6.1	10.2	14.1	12.1	12.3
Mean	29.4	29.4	31.0	32.0	31.9	32.7
Educational attainment						
No education	48.3	45.2	33.2	30.7	48.4	47.2
Primary incomplete	17.8	19.1	23.5	21.9	20.5	19.2
Primary complete	13.3	11.8	13.2	13.3	14.7	15.2
Secondary incomplete	16.7	18.9	24.5	27.6	13.5	15.7
Secondary and higher	4.0	5.0	5.6	6.5	2.8	2.7

Note: Base = Base line, End = Endline.

### Changes in use of contraceptive methods

The icddr,b evaluation found improvements in the following reproductive health indicators. The urban slum area had the highest contraceptive prevalence rate for all methods among the three areas; 59% at baseline and 65% at endline. The contraceptive prevalence rate for any modern method increased significantly from baseline to endline in Dhaka (51% to 58%) and in Nabiganj (20% to 30%). However, there was little change in contraceptive prevalence rate in Raipur (43% to 44%). In all areas significantly higher proportions of women used injectable contraceptive method at endline compared to baseline. In all areas oral pills were the most commonly used contraceptive method (Table 2).

**Table 2:** Use of contraceptive methods by women, by study areas

	**Dhaka**		**Raipur (%)**		**Nabiganj (%)**	
	**Base n = 5477**	**End (n = 5,670)**	**Base (n = 6,433)**	**End (n = 6,211)**	**Base (n = 6,288)**	**End (n = 6,419)**
All methods	58.7	64.9*	49.9	50.2	22.3	33.7*
Any modern method	51.1	58.7*	43.3	44.5	19.7	30.1*
Female sterilization	0.5	3.4	0.2	2.4	0.1	2.7
Male sterilization	0.1	0.9	0.01	0.3	0.01	1.0
Pill	33.9	29.3	28.6	22.3	14.4	18.4
IUD	0.7	0.5	1.3	1.2	0.6	0.6
Injectables	9.4	17.4*	10.6	15.1*	2.4	4.2
Implants	1.8	1.7	0.7	1.0	1.2	1.4
Condom	4.7	5.5	1.9	2.1	1.0	1.7
Any traditional methods	7.6	6.2	6.6	5.7	2.6	3.6
Periodic abstinence	5.2	3.6	4.0	3.2	1.8	2.3
Withdrawal	2.0	2.2	1.7	1.8	0.5	1.0
Herbal	0.4	0.4	0.9	0.7	0.3	0.3

Note: *Shows statistically significant difference between baseline and endline (p < .001).

**Figure 1:**
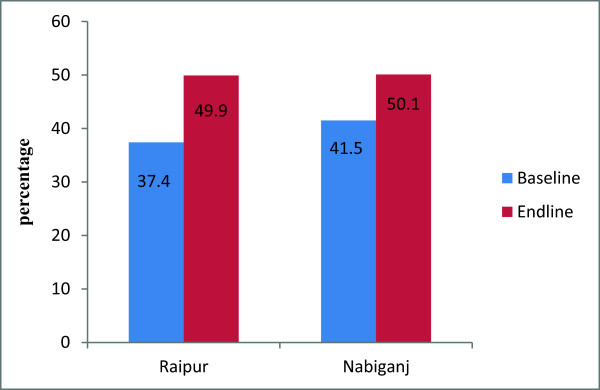
**Public sector as the source of obtaining contraceptive methods, by two rural areas.** Note: Significant difference found between baseand and endline both in Raipur and Nabiganj (p<0.001).

**Figure 2:**
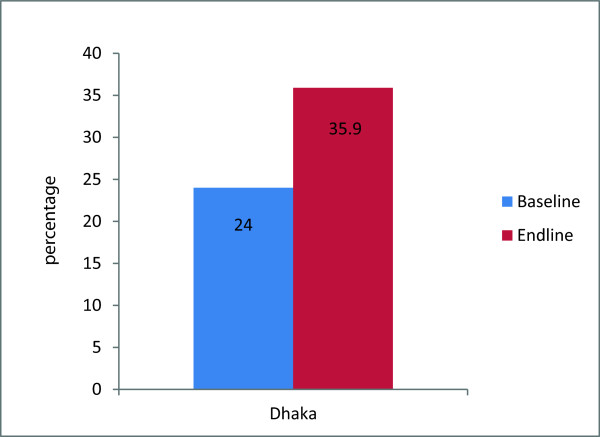
**NGO sector as the source of obtaining contraceptive methods, by urban area.** Note: Significant difference found between Baseline and Endline in Dhaka (P<0.001).

**Figure 3:**
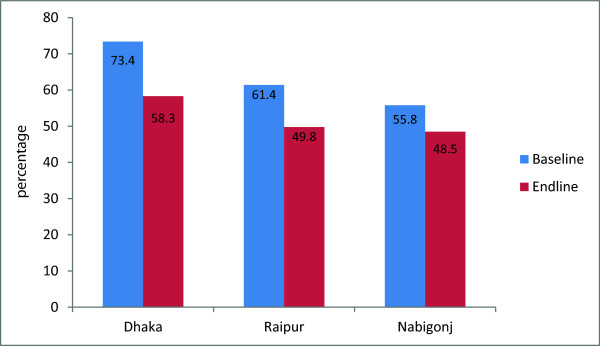
**Private sector as the source of obtaining contraceptive methods, by rural and urban areas.** Note: Significant difference found between baseline and Endline in Dhaka,Raipur, and Nabigonj (p<0.001).

In the rural areas, a significantly higher proportion of women at endline compared to baseline obtained contraceptive methods from the public sector; in Raipur, this increased 37-50% (p < .001),while in Nabiganj, it increased from 41% to 50% (p < .001) (Figure 1). Similarly in urban areas a significantly higher proportion of women obtained contraceptive methods from NGO sectors at endline compared to baseline (20% to 36%) (p < .001) (Figure 2). In all areas, use of private sector as last source of obtaining contraceptive methods has reduced at endline compared to baseline (Figure 3).

### Changes in utilization of ANC and PNC services

In Nabiganj a significant higher proportion of women at end line (57%) compared to baseline (43%) received any antenatal care (ANC). Similarly, in Raipur a significant higher proportion of women at endline (81%) compared to baseline (78%) received ANC during their last pregnancy. Such changes were not observed in the urban slum. In all areas, a higher proportion of women at endline compared to baseline received post-natal care (PNC) (p < .001) (Table 3).

**Table 3:** Status of ANC and PNC services received by women, by study area

**Outcome**	**Dhaka (%)**		**Raipur (%)**		**Nabiganj (%)**	
	**Base n = 5,477**	**End n = 5,670**	**Base n = 6,433**	**End n = 6,211**	**Base n = 6,288**	**End n = 6,419**
Received ANC	82.5	80.8	77.9	80.6*	43.9	57.1*
Received PNC	37.1	89.8*	39.7	89.0*	33.7	87.3*

Note: *Shows statistically significant difference between baseline and endline (p < .001).

### Women’s knowledge on maternal complications and utilization of qualified birth attendants during delivery

**Figure 4:**
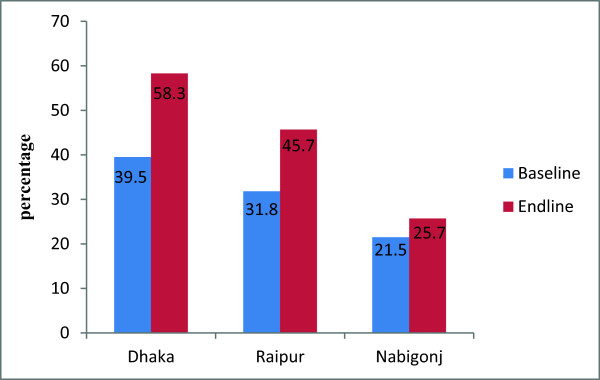
**Utilization of qualified birth attendants during delivery, by urban and rural areas.** Note: Significant difference found between baseline and Endline in Dhaka, Raipur, and Nabigonj (p<0.001).

The most commonly mentioned life-threatening complications during delivery were mal presentation, prolonged or obstructed labor (Table 4). Significantly higher proportions of women, in end line compared to baseline, mentioned delivery related life threatening complications like: mal presentation and prolonged labour in Dhaka and Nabiganj. While significant higher proportion of women knew, at endline compared to baseline, in Raipur about obstructed labor. However, only one percent or less proportion of women in all areas mentioned excessive bleeding at delivery. Table 3 also shows women’s knowledge of life-threatening complications after childbirth. Higher proportions of women in end line compared to baseline mentioned about life threatening complications after child birth; delay placental expulsion, heavy vaginal bleeding, and hypertension irrespective of study areas. Irrespective of study areas, significant higher proportions of women utilized qualified birth attendants at endline compared to baseline (Figure 4).

**Table 4:** Women’s perception on maternal complications, by study areas

**Perceived delivery related complications**	**Dhaka %**		**Raipur %**		**Nabigonj %**	
	**Base n = 1516**	**End n = 1456**	**Base n = 1829**	**End n = 1523**	**Base n = 2502**	**End n = 2174**
Mal-presentation	28.8	55.6*	49.3	45.7	35.4	43.3*
Prolonged labour	20.3	38.3*	24.8	27.4	19.5	32.7*
Obstructed labour	21.8	24.6	30.5	24.4*	24.5	35.4*
Heavy bleeding	0.5	0.1	0.4	0.4	0.3	0.1
Tetanus	0.5	1.3	1.1	2.7	4.1	0.7
Advance breaking of liquor (*“pani uge bhange”)*	0.2	0.3	0.4	0.9	0.4	0.1
Hypertension	0.3	0.3	0.3	0.7	0.8	0.2
Do not know	36.4*	6.0	19.0	10.9	33.2	6.2*
Perceived complications after delivery						
Delay in placental expulsion/no expulsion	30.5	55.8*	44.0	48.9*	29.1	56.2*
Tear in uterus	5.2	13.0*	10.5	12.1	2.0	8.8*
Heavy vaginal bleeding	19.3	34.1*	39.2	25.9*	8.0	17.7*
Fever with foul smelling vaginal discharge	0.7	2.7	1.3	1.7	0.8	1.0
Eclampsia	0.2	0.5	0.3	0.5	2.6	0.2
Tetanus	3.6*	1.2	3.6	4.6	5.9	4.1
Hypertension	2.5	9.1*	2.0	18.2*	2.6	15.2*
Anaemia	0.2	0.1	0.4	0.8	0.7	0.5
Do not know	41.6	8.9*	18.4	10.8*	49.7*	9.6

Note: *Shows statistically significant difference between baseline and endline (p < .001) (multiple responses considered).

### Utilization of health care facilities in last three months

Significantly higher proportions of women in all areas reported in end line compared to baseline that they visited health facility in last three months. In rural areas, significantly higher proportions of women in end line compared to baseline went to Government Satellite clinics, which is a public facility. In urban areas, significantly higher proportion of the women reported visiting NGO clinics at endline compared to baseline, which was expected because intervention was implemented through NGOs in urban areas (Table 5).

**Table 5:** Utilization health facilities by women in last 3 months, by area

	**Dhaka**		**Raipur**		**Nabigonj**	
	**Base n = 5839**	**End n = 6026**	**Base n = 6752**	**End n = 6602**	**Base n = 6929**	**End n = 7009**
Proportion of women visited health facility in last three months	37.7	43.2*	37.4	46.9*	27.7	34.0*
**Types of health facilities visited**	**Dhaka**		**Raipur**		**Nabigonj**	
	**Base n = 2201**	**End n = 2601**	**Base n = 2524**	**End n = 3095**	**Base n = 1922**	**End n = 2386**
NGO Satellite clinics	14.9	20.5*	0.7	0.8	1.2	0.5
Government Satellite clinics	3.0	0.7	27.0	34.1*	3.1	14.8*
H&FWC	N/A	0.0	12.8	15.9	11.9	14.3
NGO static clinic	26.9	46.4*	0.3	0.5	2.7	1.4
Upazila Health Complex	0.4	0.4	4.4	5.5	13.6	7.2*
MCWC	0.5	0.1	0.3	0.2	0.2	0.0
RD/UD (rural/urban dispensary)	0.1	0.2	0.1	0.1		0.3
Government hospital	2.7	3.5	1.3	0.9	3.0	1.1
Private clinic	14.5	10.7*	4.8	11.9*	24.6	24.2
EPI center	21.6	0.4	54.0	24.7*	16.0	28.8*
Pharmacy	21.6	25.6*	7.4	18.5*	19.3	12.7*
Others	1.2	0.3	0.9	1.7	6.7	1.5

Note: *Shows statistically significant difference between baseline and endline (p < .001).

### Knowledge on STI, reported symptoms and action taken

Overall, 9.5% to 23.8% of the surveyed women knew about any STI irrespective of timing of survey and areas. Higher proportions of women from Dhaka (23.8 in baseline versus 18.5% in endline) and Raipur (19% in endline versus 17% in endline) reported in endline compared to baseline that they had heard about STI. Proportion of women knew about specific disease like gonorrhoea and syphilis varied in different areas and changes between baseline to endline were not statistically significant (not shown in the table). Women reported experiencing STI related symptoms like burning pain during urination, sore/ulcer in the genital areas (Table 6). Practices like consultation with non qualified and indigenous providers are still prevailing at endline in all study areas, for instance, about 3% women consulted Kabiraj (traditional healer) for such problems in Nabiganj. Self treatments like washing/cleaning and taking extra amount of water were practices by many women for STI problems. However, of those who experienced such problems, higher proportions consulted doctors after their latest complication at end line compared to baseline in Dhaka.

**Table 6:** Perceived STI/RTI related symptoms by women in the last year

**STI related symptoms**	**Dhaka**		**Raipur**		**Nabigonj**	
	**Base n = 5838**	**End n = 6026**	**Base n = 6752**	**End n = 6602**	**Base n = 6928**	**End n = 7009**
Sore/ulcer in genital area	8.2	7.6	12.0	13.1	6.5	5.2
Burning during urination	13.8	6.3	22.3	18.6	9.3	4.6
Pain at the time of sexual intercourse	4.2	2.7	7.5	3.6	2.6	1.7
Excessive per vaginal bleeding	4.2	2.9	10.7	6.9	3.2	1.2
Did not have such problems	76.8	84.8	64.6	72.2	83.8	91.0
**Action taken for reported STI symptoms**	**Dhaka**		**Raipur**		**Nabigonj**	
	**Base n = 1354**	**End n = 903**	**Base n = 2487**	**End n = 1840**	**Base n = 1135**	**End n = 631**
Consulted doctors	22.2	29.2*	24.6	20.3	28.4	31.2
Visited to hospital/clinics	5.2	9.6	6.4	5.8	5.6	6.3
Consulted pharmacist	9.8	12.0*	12.3	14.4	8.9	13.9
Consulted *kabiraj*(traditional healer)	1.7	1.8	2.6	2.4	2.6	3.3
Consulted village doctor	0.4	1.0	11.1	14.0	5.4	7.9
Consulted homeopath doctor	1.5	2.3	2.0	1.8	1.1	2.4
Consulted *peercp fakir*(religious healer)	1.0	0.1	2.4	0.1	0.5	0.3
Have taken extra amount of liquids, drinks	3.2	0.2	2.0	0.0	0.7	0.0
Used hot water/*detol* to wash genitalia	5.7	8.4	3.5	5.3*	2.1	4.1
Others	0.5	0.3	0.6	0.2	0.7	0.3
Nothing was done	50.2	38.0*	32.4	36.4	44.4	28.5*

Note: *Shows statistically significant difference between baseline and endline (p < .001).

## Discussion

The evaluation of the DBRHCP interventions was to assess user perspectives and changes in key reproductive health indicators including the contraceptive prevalence rate, ante natal care, post natal care, utilization of qualified birth attendants, and health care utilization by women. This suggests that the efforts to improve existing services positively affected selected knowledge and practice related outcomes among married women of reproductive age in the low performing communities. Quayyum and colleagues [14] also reported that community level interventions can have positive impact on utilization of maternal health care in Bangladesh [14].

Two out of the three project areas showed a statistically significant increase in the use of contraceptive methods by women of reproductive age. What is more encouraging is that the injectable method of contraception, which is a semi-permanent method, increased in all three areas. This is leading to a better ‘method mix’ that might help achieve replacement level fertility in the long term. Both the public (in rural areas) and the NGO providers (in urban areas) played an active role in counseling for uptake of the appropriate method. A study done in rural Bangladesh have shown that although factors like age, number of living children, having a male child, women’s education, religion, and NGO membership were important determinants of contraceptive use and method choice, discussion between husband and wife on family planning was most influential single factor for contraceptive use and selection of method [15]. Therefore couple counseling for increased uptake of contraceptive methods and method choice appears to be very crucial.

As reported by the present study, ANC service utilization increased at the end of the project period which possibly has contributed in increased use of qualified birth attendants at the endline. A study done in Bangladesh found that there is an association between ANC visits with increased uptake of facility-based delivery and peri-natal survival [16]. With the significant increase in PNC in all project areas, more mothers were educated on post-partum contraception and birth spacing. Through uptake of a modern method during post-partum period, women might be able to delay the next conception.

Although the present study found that generally more women at endline compared to baseline knew about maternal complications, still they had low level of awareness about important complications like heavy bleeding at the time of delivery. However, a considerable proportion of women identified post partum heavy bleeding as a serious complication after child birth. A national survey done in Bangladesh reported that despite almost half of women reported having one or more complications during pregnancy that they perceived as life threatening, only one in three sought treatment from a qualified provider [5]. Pembe et al. [17] found that having secondary or higher education lead to a six fold increase of awareness on obstetric danger signs in comparison to having no education. This study also reported that awareness on maternal complications increased significantly by increasing age of the mother, number of deliveries, number of antenatal visits, whether the delivery took place at a health institution and whether the mother was informed of having a risks/complications during antenatal care [17].

The present study reported that overall awareness on STIs were low among surveyed women even at endline. A study done in India also reported that despite of knowing importance of formal medical care for STIs, many women consulted traditional healers or dependent on home remedies for STI problems [18]. The present study found that consultation with indigenous practitioners and self treatment were practices for STI problems even at the end of the project period. Possibly women felt more comfortable seeking care from non formal providers in terms of privacy, confidentiality or they found it less costly which must be explored by future studies. As indicated by Sihavong [19], women might not avail treatment for STI problems due to lack of privacy and confidentiality at the facility level [19]. Khan and colleagues [20] found that awareness of STIs was most strongly and positively associated with the education of the women and their husbands, women’s mobility, and attendances in mothers’ clubs [20]. Therefore, more investment in women’s formal education as well as women’s participation in educational sessions is found to be very beneficial in raising their awareness on such sensitive issues. However, awareness creation is not enough, health care facilities should offer STI related services with adequate privacy and confidentiality to attract women in obtaining services when required.

The present study had a few limitations. Since there was no control area and the present study adopted only a pre-post evaluation approach, it is possible that these improvements were part of broader secular trends. However, given the slow improvements in RH indicators in the low performing areas of Bangladesh over the past decade [2], this is unlikely. As we observed in the latest demographic survey, total fertility rates (TFR) remained high in two Divisions; 3.1in Sylhet and 2.8 in Chittagong, compared to the national rate of 2.3 [21]. Another limitation of the study is intervention period was relatively short to capture impacts at practice level.

The incremental program costs of the various components under this project have been collected and a cost estimation exercise has been done (unit cost of 3.38 USD per beneficiary) with a view to scaling up the DBRHCP [22]. The study findings suggest that investment in the reproductive health sector through government programs positively improves community reproductive health outcomes. Therefore, the Government might consider allocating resources to scaling up such models in similar low performing settings.

## Conclusions

The improvements in several important RH indicators in the intervention areas suggest that the interventions affected selected outcomes reported in the study. These findings can be used to develop appropriate strategies for improved reproductive health service delivery that are demand-based, effective and replicable in the national program. Without considerable modification to make reproductive health a client orientated service, Bangladesh is unlikely to achieve replacement level fertility in the near future. Continued systematic evaluations of innovative programs are required to improve reproductive health outcomes particularly in low performing areas of Bangladesh.

## Competing interests

The authors declare that they have no competing interests.

## Authors’ contribution

RG developed the concept, designed the study, provided overall guidance to the study team, analyzed data, and drafted the manuscript. HK was involved in field implementation of the study, data quality control, and manuscript writing. Saha NC contributed in data management, data analysis and provided assistance in manuscript writing. All authors read and approved the final manuscript.

## Pre-publication history

The pre-publication history for this paper can be accessed here:

http://www.biomedcentral.com/1471-2458/14/478/prepub
